# A survey on infection management practices in Italian ICUs

**DOI:** 10.1186/cc11866

**Published:** 2012-11-15

**Authors:** Matteo Bassetti, Raffaele De Gaudio, Teresita Mazzei, Giulia Morace, Nicola Petrosillo, Pierluigi Viale, Giuseppe Bello, Sofia La Face, Massimo Antonelli

**Affiliations:** 1 Infectious Diseases Department, Santa Maria Misericordia University Hospital, Piazzale Santa Maria della Misericordia 15, 33100 Udine, Italy; 2 Department of Critical Care, Anesthesiology and Intensive Care Section, University of Florence, Largo Brambilla 3, 50134 Firenze, Italy; 3 Department of Preclinical and Clinical Pharmacology, University of Florence, Via delle Oblate 1, 50141 Firenze, Italy; 4 Department of Public Health, Microbiology, Virology, University of Milan, Via Pascal, 36, 20133 Milano, Italy; 5 Second Division for Infectious Diseases, Lazzaro Spallanzani National Institute for Infectious Diseases, Via Portuense 292, 00149 Roma, Italy; 6 Department of Medical and Surgical Sciences, Alma Mater Studiorum University of Bologna, Via Albertoni 15, 40138 Bologna, Italy; 7 Department of Intensive Care and Anesthesiology, Agostino Gemelli Medical Center, Catholic University of the Sacred Heart, Largo Agostino Gemelli 8, 00168 Roma, Italy; 8 Anti-infective and Transplant Team, Specialty Care Medical Affairs, Pfizer Italia S.r.l., Via Valbondione, 113, 00188 Roma, Italy

## Abstract

**Introduction:**

An online survey was conducted to characterize current infection management practices in Italian intensive care units (ICUs), including the antibacterial and antifungal drug regimens prescribed for various types of infections.

**Methods:**

During February and March 2011, all 450 ICUs in public hospitals in Italy were invited to take part in an online survey. The questionnaire focused on ICU characteristics, methods used to prevent, diagnose, and treat infections, and antimicrobials prescribing policies. The frequency of each reported practice was calculated as a percentage of the total number of units answering the question. The overall response rate to the questionnaire was 38.8% (175 of the 450 ICUs contacted) with homogeneous distribution across the country and in terms of unit type.

**Results:**

Eighty-eight percent of the responding facilities performed periodical surveillance cultures on all patients. In 71% of patients, cultures were also collected on admission. Endotracheal/bronchial aspirates were the most frequently cultured specimens at both time points. Two-thirds of the responding units had never performed screening cultures for methicillin-resistant *Staphylococcus aureus*. Around 67% of the ICUs reported the use of antimicrobial de-escalation strategies during the treatment phase. In general, the use of empirical antimicrobial drug regimens was appropriate. Although the rationale for the choice was not always clearly documented, the use of a combination therapy was preferred over antibiotic monotherapy. The preferred first-line agents for invasive candidiasis were fluconazole and an echinocandin (64% and 25%, respectively). Two-thirds of the ICUs monitored vancomycin serum levels and administered it by continuous infusion in 86% of cases. For certain antibiotics, reported doses were too low to ensure effective treatment of severe infections in critically ill patients; conversely, inappropriately high doses were administered for certain antifungal drugs.

**Conclusions:**

Although infection control policies and management practices are generally appropriate in Italian ICUs, certain aspects, such as the extensive use of multidrug empirical regimens and the inappropriate antimicrobial dosing, deserve careful management and closer investigation.

## Introduction

Drug prescribing patterns and clinical practices differ from country to country, but variation has also been observed among and within medical specialties. The differences are probably related to the needs of different patient populations, but they also reflect culture-specific and individual attitudes and forms of decision making among physicians [1]. Among developed countries, for example, Italy has one of the highest rates of antibiotic use [2]. Throughout the world, community use of these drugs appears to be significantly conditioned by patients' demand and other social factors [1,3]. These type of influences should be virtually nonexistent in intensive care units (ICUs), where antimicrobial therapy is usually prescribed empirically on the basis of limited evidence [4,5]. When compared with general medical wards, ICUs displays higher rates of antimicrobial use because their patients are at higher risk for severe nosocomial infections [6,7]. It has been estimated that over 50% of all critically ill patients will receive at least one antibiotic during their ICU stay, in most cases for pneumonia [8-10]. Widespread, often inappropriate, use of antimicrobials can favor the emergence of resistances [10,11] and increase the occurrence of treatment failure, toxicity, mortality rates as well as the rise in the costs of care among critically ill patients [10,12].

The aim of the present study was to characterize the current infection management practices in Italian ICUs, including the characterization of the antibacterial and antifungal drug regimens prescribed for various types of infections and the collection of evidence-based information support in the intensive care setting. We believe that the educational intervention programs carried out in Italy over the last few years by local professional associations have improved the infection control policies, the management practices, and the prescribing patterns in ICUs.

## Materials and methods

Institutional review board approval was waived for this email-based survey since it did not involve collection of data on individual patients. The survey questionnaire was prepared by a panel of experts in the fields of intensive care, infectious diseases, pharmacology, and microbiology and endorsed by the Società Italiana di Anestesia Analgesia Rianimazione e Terapia Intensiva (SIAARTI, Italian Society of Anesthesia and Intensive Care) and organized in different sections. Section I focused on the ICU's characteristics (for example, location, number of beds, level of care, type of unit/hospital). Section II contained questions (mostly multiple-choice) on the approaches used to diagnose and treat infections (for example, use and characteristics of surveillance cultures, screening procedures) and infection management practices (for example, consultation of infectious disease specialists, access to antimicrobial susceptibility test, prescribing policies for antibiotics and antifungal drugs).

During the months of February and March 2011, the SIAARTI sent emails to all physicians working in the 450 ICUs located in public hospitals in Italy, inviting them to participate in the survey. The email explained the purpose of the survey, the information handling practices, and provided instructions for online completion of the questionnaire. Each ICU director who accepted the invitation was given password-controlled access to the survey and was responsible for collecting all the information from the physicians working in the unit to ultimately fill in one questionnaire reflecting the overall opinion and practices. Responses submitted between 30 April and 2 June 2011 were collected blindly into a central database and analyzed.

Submitted questionnaires were excluded from the analysis only if Section I had not been completed. Failure to answer one or more questions in Section II was not a cause for exclusion. In Section II, the frequency of each reported practice was calculated as a percentage of the total number of units that answered the question regarding that practice.

## Results and discussion

### Characteristics of participating ICUs

As summarized in Table 1, 175 of the 450 ICUs responded to the questionnaires, and they all met the inclusion criteria for data analysis (overall response rate: 38.8%). Seventy-five (43%) participating units were located in northern Italy, 56 (32%) in central Italy, and 44 (25%) in southern regions. Most were located in small (< 500 beds, 54%) or medium-sized (500 to 1000 beds, 30%) hospitals. Sixty-three (36%) were at teaching hospitals. Eight-four percent were polyvalent ICUs, 13% were surgical units, and other unit types accounted for the remaining 3%. Most units (*n *= 163, 93%) contained four to twelve beds. The overall number of admissions per year varied from 80 to 1600, although in 63% of the units the range was much narrower (100 to 400 per year). The average ICU stay reported by respondents ranged from one to fifteen days (mean eight days).

**Table 1:** Sample characteristics of the ICU center respondents.

Response rate	175/450 (38.8%)
**Geographical distribution of ICU respondents**	

Northern Italy	75/175 (43%)
Central Italy	56/175 (32%)
Southern Italy	44/175 (25%)

**Type of hospital**	

Teaching hospital	63/175 (36%)
Non-teaching hospital	112/175 (64%)

**Hospital size**	

< 500 beds	85/175 (49%)
500-1000 beds	51/175 (29%)
1000-1500 beds	15/175 (8%)
< 1500 beds	24/175 (14%)

**Type of ICU**	

Surgical	28/175 (16%)
Mixed (Medical/surgical)	147/175 (84%)

**Number of ICU beds**	

1-6	77/175 (44%)
7-10	58/175 (33%)
11-18	33/175 (19%)
19-50	7/175 (4%)

### Infection control and management practices

Responses to infection control policies are summarized in Tables 2, 3 and 4. Table 1 shows responses to questions regarding practices and resources used for the diagnosis, prevention, and management of infections, which were answered by 146 to 162 of the 450 ICUs contacted (response rate from 32% to 36%). Information on routine empirical drug regimens used for specific types of infections is shown in Tables 1 and 2 (response rate from 32% to 38%). Table 3 shows how major antibacterial and antifungal drugs were prescribed in terms of doses, schedules, and methods of administration (response rate from 20% to 33%). The number, types, and location of the 175 units that took part in our survey are quite representative of the 450 ICUs that are present in our country. The characteristics of the participating units are also comparable to those documented in the annual report of the Italian Group for the Evaluation of Interventions in Intensive Care [13].

**Table 2:** Infection control and management practices in participating ICUs.

Practices	Use - number (%)^a^
Surveillance cultures	
○ on ICU admission	104/146 (71)
○ after ICU admission	128/146 (88)

Regular reports on antimicrobial resistance profiles of organisms isolated in the hospital	66/151 (41)

Automatic lab alerts when high-risk isolates are recovered^b^	88/149 (59)

Nasal-swab screening for MRSA and decolonization when cultures are positive	
○ Yes	24/151 (16)
○ No	103/151 (68)
○ In selected cases	24/151 (16)

Quantitative cultures of tracheobronchial secretions	108/156 (69)

Samples cultured to diagnose pneumonia	
○ endotracheal aspirate	79/162 (49)
○ bronchoalveolar lavage fluid	68/162 (42)
○ protected-specimen brush	15/162 (9)

Routine use of the Candida colonization index	44/148 (30)

Use of the procalcitonin assay	100/152 (66)

Consultation of infectious disease specialists for infection management	
○ Routinely	16/149 (11)
○ In selected cases	82/149 (55)
○ Rarely	36/149 (24)
○ No	15/149 (10)

Use of antibiotic combinations for first-line empirical treatment of specific infections	
○ Community-acquired peritonitis	92/162 (57)
○ Early-onset ventilator-associated pneumonia (VAP)	107/162 (66)
○ Late-onset ventilator-associated pneumonia (VAP)	142/162 (88)
○ Community-acquired pneumonia (CAP)	113/162 (70)
○ Hospital-acquired pneumonia (HAP)	128/162 (79)
○ Health-care-associated pneumonia (HCAP)	138/162 (85)
○ Post-surgical peritonitis	133/162 (82)
○ Catheter-related bacteremia	107/162 (66)
○ Purulent meningitis	120/162 (74)

Therapy de-escalation when culture results are back	109/162 (67)

^a^Denominators represent the number of ICUs that answered the specific question; ^b^high-risk isolates include MRSA, vancomycin-resistant enterococci, extended-spectrum beta-lactamase-producing gram-negative bacilli, *Candida *spp., and so on). MRSA, methicillin-resistant *Staphylococcus aureus*.

**Table 3:** Antimicrobials most commonly used for empirical treatment of specific infections.

Infection/Drugs	Frequency **number (%)**^a^	Infection/Drugs	Frequency **number (%)**^a^
**Early VAP (<5 days)**		**Suspected catheter-related bacteremia**	
Piperacillin/tazobactam Levofloxacin Ceftriaxone	37/146 (25) 25/146 (17) 19/146 (13)	Vancomycin Piperacillin/tazobactam Meropenem	70/171 (41) 21/171 (12) 15/171 (9)

**Late VAP (≥5 days)**		**Suspected candidemia and/or invasive**	
Piperacillin/tazobactam Meropenem Ceftazidime	50/142 (35) 23/142 (16) 17/142 (12)	**candidiasis** Fluconazole Caspofungin Voriconazole	97/151 (64) 32/151 (21) 9/151 (6%)

**Community-acquired pneumonia**		**Community-acquired peritonitis**	
Ceftriaxone Levofloxacin Amoxicillin/clavulanic acid	42/156 (27) 31/156 (20) 23/156 (15)	Piperacillin/tazobactam Ampicillin/sulbactam Cefotaxime	37/161 (23) 26/161 (16) 16/161 (10)

**Hospital-acquired pneumonia**		**Postoperative peritonitis**	
Piperacillin/tazobactam Levofloxacin Ceftriaxone	51/171 (30) 26/171 (15) 22/171 (13)	Piperacillin/tazobactam Meropenem Ertapenem	63/165 (38) 46/165 (28) 8/165 (5)

**Health-care-acquired pneumonia**		**Purulent meningitis**	
Piperacillin/tazobactam Meropenem Ceftazidime	39/143 (27) 24/143 (17) 13/143 (9)	Ceftriaxone Linezolid Cefotaxime	111/156 (71) 6/156 (4) 6/156 (4)

^a^Denominators represent the number of ICUs that answered the specific question. VAP, ventilator-associated pneumonia.

**Table 4:** Most frequently used doses and schedules of antibiotic and antifungal drugs.

Drug/Administration	Frequency Number (%)^a^	Drug/Administration	Frequency Number (%)^a^
**Vancomycin**		**Ceftazidime**	
TDD: 2 g	104/142 (73)	TDD: 6 g	89/141 (63)
Infusion: Continuous	122/142 (86)	3 g	20/141 (14)
Other: TDM	94/142 (66)	Infusion: Continuous	68/141 (48)
		Schedule: 3 doses/day	64/141 (45)

**Teicoplanin**		**Ceftriaxone**	
TDD: 400 mg	43/110 (39)	TDD: 2 g	72/141 (51)
800 mg	32/110 (29)	4 g	45/141 (32)
Loading dose	86/90 (96)	Schedule: 2 doses/day	78/141 (55)
		Once daily	64/141 (45)

**Piperacillin/tazobactam**		**Cefepime**	
TDD: 18.0 g	61/148 (41)	TDD: 6 g	51/141 (36)
13.5 g	41/148 (28)	4 g	28/141 (20)
Infusion: Intermittent	61/148 (41)	Schedule: 3 doses/day	75/141 (53)
Continuous	55/148 (37)	2 doses/day	66/141 (47)
Prolonged (3-4 hours)	33/148 (22)		

**Meropenem**		**Levofloxacin^b^**	
TDD: 3 g	62/138 (45)	TDD: 1000 mg	81/139 (58)
6 g	26/138 (19)	750 mg	33/139 (24)
Infusion: Prolonged intermittent	65/138 (47)	Schedule: 2 doses/day	90/139 (65)
Intermittent infusion	55/138 (40)	Once daily	49/139 (35)
Continuous infusion	18/138 (13)		

**Imipenem**		**Ciprofloxacin^b^**	
TDD: 2 g	66/135 (49)	TDD: 1200 mg	61/138 (44)
3 g	28/135 (21)	800 mg	44/138 (32)
Schedule: 4 doses/day	99/135 (73)	Schedule: 2 doses/day	70/138 (51)
3 doses/day	36/135 (27)	3 doses/day	68/138 (49)

**Amikacin**		**Fluconazole**	
TDD: 1 g	65/120 (54)	TDD: 400 mg	77/137 (56)
1.5 g	13/120 (11)	800 mg	25/137 (18)
Schedule: Once daily	106/120 (88)	Schedule: Once daily	104/137 (76)
		2 doses/day	33/137 (24)
		Loading dose	114/137 (83)

**Gentamicin**		**Voriconazole**	
TDD: 240 mg	35/120 (29)	TDD: 8 mg/kg	56/137 (41)
160 mg	18/120 (15)	4 mg/kg	27/137 (20)
Schedule: Once a day	106/120 (88)	Loading dose	125/137 (91)

**Daptomycin**		**Anidulafungin**	
TDD: 6 mg/kg	70/137 (51)	TDD: 100 mg	94/124 (76)
8 mg/kg	29/137 (21)	Loading dose	119/124 (96)

**Linezolid**		**Caspofungin**	
TDD: 1200 mg	128/132 (97)	TDD: 50 mg	114/127 (90)
Infusion: Intermittent	111/132 (84)	Loading dose	126/127 (99)

**Tigecycline**		**Micafungin**	
TDD: 100 mg	130/134 (97)	TDD: 100 mg	105/128 (82)
Schedule: 2 doses/day	133/134 (99)		
Loading dose	131/134 (98)		

**Colistin**		**Amphotericin B**	
Intravenous		TDD:	
TDD: 6,000,000 IU	29/107(27)	Liposomal	
9,000,000 IU	27/107 (25)	3 mg/kg	61/127 (48)
Schedule: 3 times/day	65/107 (61)	5 mg/kg	48/127 (38)
Loading dose	48/107 (45)	Lipid complex	
Aerosol		5 mg/kg	54/101 (53)
TDD: 3,000,000 IU	36/107 (34)	3 mg/kg	29/101 (29)
Schedule: 3 times/day	61/107 (57)		

^a^Denominators represent the number of ICUs that answered the specific question, ^b^levofloxacin and ciprofloxacin are the only fluoroquinolones available in Italy. TDD, total daily dose (intravenous unless otherwise stated); TDM, therapeutic drug monitoring.

### Practices and resources used for the diagnosis, prevention, and management of infections

Although the importance of surveillance cultures for detecting carriers of drug-resistant microorganisms, improving nosocomial infection control, and guiding empirical antimicrobial treatment in ICUs has been documented [14,15], there are conflicting opinions that routine surveillance cultures are indicated in critically ill patients [16].

In our study, we found that 88% of the participating units periodically performed surveillance cultures on all patients, and in 71% of cases the cultures were also collected on admission (Table 2). Endotracheal and bronchial aspirates were the most frequently cultured specimens at both time points (92% and 90% of the units, respectively), which reflects a common concern among Italian intensivists of prevention and prompt detection of pneumonia. Regarding methicillin-resistant *Staphylococcus aureus *(MRSA) surveillance, although there are studies to support the use of active surveillance for high-risk patients [17,18], there is not sufficient evidence to justify the mandatory use of this control measure [19]. Screening programs for MRSA colonization are expensive and, for certain authors, of dubious utility. Universal screening of large populations is not cost-effective, whereas targeted screening of high-risk populations may deserve additional study [20]. In our survey, two-thirds of the responding units never tested for MRSA colonization. This finding was consistent with the percentages of units that reported to collect nasal swabs at the time of the admission (46%) and after ICU admission (22%). Although the potential role of Candida colonization index for early detection of candidemia remains controversial, two recent Italian reports support the value of this tool [21,22]. In the survey, we observed that very few units used the Candida colonization index during the patients' admission assessment (3%), and fewer than 20% made regular use of the index after admission.

Over half of the participating sample (88 ICUs) received automatic alerts when multidrug-resistant (MDR) or sentinel microorganisms were isolated. The majority of the units were located in large and/or university hospitals in northern or central Italy. The geographic distribution of the hospitals that provided this service reflects regional rather than national rules. Access to information on local patterns of drug susceptibility was reported only in around 40% of the participating units. In roughly half of these cases (33/66), the data were updated every six (33%) or twelve (24%) months. Only three of the 66 ICUs mentioned (4.3%) had access to weekly updates. In our opinion, these reports need to be issued at least once a month, even if no MDR microorganisms have been isolated, to increase physicians' awareness of the local microbial epidemiology. This seems particularly important in light of the low number [16/149 (11)] of units that regularly consulted an infectious disease specialist for advice on antimicrobial use (Table 2). Two-thirds of the responding units reported the use of the procalcitonin assay. Although procalcitonin seems to be a valuable predictor of bacterial infections and an effective tool for reducing unnecessary antibiotic use [23,24], its universal use in critically ill patients might have negative consequences [25,26]. Around 67% of the centers reported using de-escalation strategies during antibiotic treatment. (In this case - and others - the possibility of discrepancies between self-perceived and actual behaviors cannot be excluded). Although a recent Cochrane review found insufficient evidence to recommend for or against antimicrobial de-escalation in adults with a diagnosis of sepsis [27], this strategy appears theoretically correct and capable of promoting therapeutic appropriateness.

### Empirical antimicrobial regimens

It is complex to comment on the appropriate use of empirical antibiotic therapy given the wide variation of resistance amongst different ICUs. However, the empirical regimens used in Italian ICUs to treat the most common infections appear - on the whole - appropriate, as summarized by Table 3. It is noteworthy, however, that more than half the participating units (57% to 88%) used drug combinations rather than monotherapy for all types of infection (Table 2) and, particularly, in cases of pneumonia and postoperative peritonitis. Several original reports and systematic reviews have raised questions regarding the benefits of combination therapy (which generally includes at least one beta-lactam antibiotic) for serious bacterial infections in immunocompetent and immunocompromised patients [28-30] .

The frequent use of vancomycin for suspected catheter-related bloodstream infections (41%) cannot be considered inappropriate. Nevertheless, increasing vancomycin minimum inhibitory concentrations (MICs) - recently defined as MIC creep - have indeed been documented in *S. aureus *isolates, but the impact of vancomycin MICs on outcomes is still being debated [31]. The rationale for treating these infections with piperacillin-tazobactam (reported in up to 12% of the ICUs) is questionable, since over 80% of these infections are caused by *Staphylococcus *spp [32].

The preferred drugs reported for community-acquired and postoperative peritonitis were beta-lactams (Table 3). It is worth noting that in 50% of the participating units piperacillin/tazobactam, which has itself an excellent antianaereobic coverage, were used in association with another antianaerobic drug, metronidazole. In numerous studies, this type of redundant coverage has failed to improve clinical outcomes, and it is also associated with adverse effects and with selection of resistant strains [33].

The preferred first-line drug for invasive candidiasis was fluconazole (64%), followed by echinocandins (25%). Several consensus statements have been published on the treatment of candidemia and invasive candidiasis. The Infectious Diseases Society of America recommends fluconazole as a single antifungal agent or an echinocandin-based therapy [34]. Therefore, in 2011, the use of fluconazole was justified for stable patients, and echinocandins were also a reasonable choice in adults.

Inadequate or delayed antifungal therapy has significantly been linked to increased mortality [35], thus it is virtually impossible to reliably predict the nature or antimicrobial susceptibility of the causative *Candida*. For these reasons, starting an empirical treatment with fluconazole may have been considered inappropriate by many Italian clinicians.

### Dosing, schedules, and administration

The data presented in Table 4 provide a picture of how antibiotics and antifungals are being administered in Italian ICUs. This is an important point since many of the pathophysiological changes associated with severe acute illness or sepsis (for example, increased capillary permeability, third spacing, increased volume of distribution, impaired renal and/or liver function) can affect antimicrobial pharmacokinetics (PK), especially those of drugs that are excreted renally (for example, beta-lactams, aminoglycosides, glycopeptides). Administration of these drugs to severely ill patients at dosages defined in studies conducted in healthy volunteers often produces suboptimal serum and tissue concentrations [36]. The method of administration is equally important. Beta-lactams, for example, are time-dependent antibiotics, and a time above MIC of 80% to 100% is required for maximum activity. Consequently, they are often administered via continuous or extended (3 to 4 h) infusion, an approach that improves efficacy and may also reduce the risk of inducing resistance [37].

In most units (132/148 - 89%), daily doses of piperacillin-tazobactam ranged from 13.5 to 18 g, and more than half the units (59%) infused the drugs over an interval longer than 3 to 4 hours. Compared with intravenous bolus delivery, extended infusion of this drug combination was recently shown to reduce mortality and shorten hospital stays in ICU patients with *Pseudomonas aeruginosa *infections [38]. Around 60% of the reporting units gave meropenem as a continuous or extended infusion. In critically ill patients at high risk for infections caused by resistant strains of gram-negative bacteria (*P. aeruginosa, Acinetobacter*), these approaches are more likely to achieve the optimal pharmacodynamic (PD) target [39] .

Several studies have emphasized the value of area under the curve (AUC)/MIC ratios for predicting the efficacy of glycopeptide-based regimens. The most commonly used daily doses of teicoplanin were 6 mg/kg and 12 mg/kg (corresponding to total daily doses of 400 and 800 mg, respectively) and almost all units (96%) administered a loading dose of this drug, which is important in ICU patients to ensure rapid achievement of therapeutically effective concentrations. Therapeutic drug monitoring (TDM) is strongly recommended during vancomycin therapy to optimize drug exposure and prevent toxicity [40]. Two-thirds of the ICUs monitored blood levels of vancomycin, which was usually administered as a continuous infusion (86%) (Table 3). However, three-quarters of the units used standard daily dosages (2 grams) that are probably too low for critically ill patients. A recent PK/PD analysis of data for 191 ICU patients found that with standard vancomycin dosages the recommended AUC 0 to 24/MIC breakpoint for *S. aureus *is rarely achieved unless the patient has renal failure or is over 65 years of age. Otherwise, the daily dose needs to exceed 3 grams. This study also emphasized the need to consider agents other than vancomycin when methicillin-resistant or glycopeptide-intermediate *S. aureus *strains are involved [41].

For the aminoglycosides, which are concentration-dependent antibiotics, the efficacy target seems to be a maximum serum drug concentration (Cmax)/MIC ratio of 10 to 12 [42]. A single daily administration of 5 to 7 mg/kg of gentamicin is currently recommended, and a similar approach can be used with amikacin (15 to 20 mg/kg/day) [43]. It is important to recall that serum levels of aminoglycosides vary widely in critically ill patients, and increased volumes of distribution may result in suboptimal peak levels [44]. Almost all the participating units gave aminoglycosides once daily, but in light of the above findings, the most frequently reported doses (15 mg/kg/day and 3 to 5 mg/kg/day for amikacin and gentamicin, respectively) are probably too low to reach the optimal PK-PD target. In contrast, levofloxacin and ciprofloxacin (the only fluoroquinolones available in Italy) were generally used at maximum recommended daily doses: levofloxacin 1000 mg (58%) in two doses or 750 mg (25%) in a single daily dose; ciprofloxacin 1200 mg (44%) or 800 mg (32%) in two to three doses. These regimens should produce an AUC/MIC ratio of about 125, which correlates with good microbiological and clinical outcomes in critically ill patients [45].

Colistin has recently re-emerged as a last-resort treatment for MDR gram-negative bacterial infections. In several PK-PD studies, its effects have proved to be concentration-dependent, and AUC/MIC and Cmax/MIC ratios are probably the factors correlated with outcome [46,47]. Around half the participating centers appear to be using this drug according to the more recent literature in terms of daily doses (6 to 9 million IUs in 52% of the cases) and after the administration of a loading dose (45%) (Table 4).

Almost all ICUs administered a fixed dose of tigecycline (100 mg loading dose followed by 50 mg every 12 h). Regarding daptomycin, preliminary evidence suggests that doses exceeding 6 mg/kg/day may be associated with more favorable outcomes [48]. Around 21% of the ICUs reported daptomycin doses exceeding the approved range (4 to 6 mg/kg/day).

Analysis of antifungal drug-prescribing practices revealed that most ICUs used appropriate daily and loading doses of both the azoles and echinocandins. However, in 38% of the participating units, liposomal amphotericin B was administered at a daily dose of 5 mg/kg despite clear evidence that doses above 3 mg/kg/day do not have an increased efficacy in the treatment of invasive fungal infections [49]. Furthermore, roughly 20% of the units reported using fluconazole at a daily dose of 12 mg/kg. This is a much higher dose than the one recommended by standard guidelines, and even when it is administered empirically in a critically ill patient with unexplained fever and a high risk for invasive candidiasis, it is unjustified and inappropriate [34].

TDM is indicated for many azoles (especially itraconazole, voriconazole, and posaconazole) [50]. Blood levels of antifungal drugs are characterized by broad, unpredictable variability related to multiple factors, including patients' age, genetic background, compliance, gastrointestinal function, use of co-medications, and liver and/or renal dysfunction [45]. Negative outcomes are associated with both antifungal underdosing (treatment failure) and overdosing (toxicity) [50]. Unfortunately, real-time measurement of antifungal drug blood levels is not routinely available, which may explain why voriconazole TDM was not mentioned by our ICUs.

Our study has several limitations. First of all, our questionnaire did not by any means explore all aspects of infection control. Second, although the sample surveyed is representative of the Italian ICUs, the overall response rate was less than 40%, with even lower response rates in certain questions. This raises the possibility of a selection bias toward ICUs that are more concerned with the efficacy of their infection management policies and procedures. Third, all of our data reflect self-reported behavior that may or may not coincide with actual practices, and physicians often tend to overestimate their use of recommended practices (tests, procedures, drug regimens) [51]. Given the last two considerations, certain problematic areas in the ICU setting may have escaped our detection. On the other hand, however, those that did emerge are likely to be realistic, and they may even underestimate more serious and widespread issues.

Our survey indicates that certain resources and tools for infection control are being underused in Italian ICUs, including MRSA screening and updated reports on local microbial epidemiology. As for antimicrobial drug therapy, prescribing practices were generally appropriate, but certain aspects deserve closer investigation, above all the widespread preference for multidrug empirical regimens, which in some cases seemed difficult to justify. Furthermore, certain antibiotics were reportedly used at doses unlikely to achieve PK/PD targets in critically ill patients, whereas inappropriately high doses were reported for certain antifungal drugs. There is little evidence that practices of this type can be corrected by guidelines, restrictive formularies, or other administrative measures, probably because they usually stem from a lack of information [52]. Our findings will hopefully serve to stimulate Italian ICUs to critically review their own infection management policies with the aim of reinforcing the strong points and correcting practices likely to be less effective.

## Conclusions

In this study, although infection control policies and management practices are generally appropriate in Italian ICUs, certain aspects, such as the extensive use of multidrug empirical regimens and the inappropriate antimicrobial dosing, deserve careful management and closer investigation.

## Key messages

• Considering the number, types, and locations of the 175 units that took part in our survey, the sample is quite representative of Italy's 450 ICUs.

• The majority of the participating units periodically performed surveillance cultures on and after admission.

• Empirical antimicrobial drug regimens for specific infections were generally appropriate. Combination therapy was preferred over monotherapy (sometimes without clear justification), and around 70% of the centers reported using de-escalation strategies during the treatment phase.

## Abbreviations

AUC: are under the curve; CAP: community-acquired pneumonia; Cmax: maximal concentration; HAP: hospital-acquired pneumonia; HCAP: health-care-associated pneumonia; ICU: Intensive Care Unit; MDR: multidrug-resistant; MIC: minimum inhibitory concentration; MRSA: methicillin-resistant *Staphylococcus aureus*; PD: pharmacodynamics; PK: pharmacokinetics; SIAARTI: Società Italiana di Anestesia Analgesia Rianimazione e Terapia Intensiva; TDD: total daily dose; TDM: therapeutic drug monitoring; VAP: ventilator-associated pneumonia.

## Competing interests

MB serves on scientific advisory boards for Pfizer Inc., Merck Serono, Novartis, Shionogi & Co., Ltd., and Astellas Pharma Inc.; has received funding for travel or speaker honoraria from Pfizer Inc., Merck Serono, Novartis, GlaxoSmithKline, Gilead Sciences, Inc., Sanofi-Aventis, Cephalon, Inc., Bayer Schering Pharma, Janssen, and Astellas Pharma Inc. RDG has received speaker honoraria from Astellas, MSD and Pfizer, serves on scientific advisory boards for MSD and Pfizer and has received research grants from Novartis, Gambro and Pfizer. TM has received honoraria from Angelini, Astellas, Eli Lilly, Janseen-Cilag, Novartis, Pfizer, Sanofi-Aventis, Schering- Plough, Valeas and Zambon. GM has received honoraria from Astellas, Merck Sharpe & Dohme, Gilead and Pfizer. NP received fees in the speakers' bureau for Pfizer, Sanofi Aventis, Gilead, Myers Squibb, Janssen Cilag, Johnson & Johnson, MSD, Novartis, and for scientific boards for Johnson & Johnson, Carefusion, MSD and Novartis. PLV has received honoraria from Astellas, Merck Sharpe & Dohme, Gilead, Pfizer, Novartis, Sanofi-Aventis, Therabel, Abbott, Viiv and BMS. GB reports no conflict of interests. SLF is employed by Pfizer, Italy and has Pfizer stock options. MA has received speaker honoraria from Pfizer, Covidien and Gilead and research grants from Pfizer and Lilly.

## Authors' contributions

MB was involved in study design, conception, coordination, data acquisition, data/statistical analyses and drafting the manuscript. RDG was involved in data acquisition, drafting the manuscript, critically revising for medical content. TM was involved in data collection and critically revising for medical content. GM was involved in data collection and critically revising for medical content. NP was involved in data acquisition and data analyses. PLV was involved in study design, conception and critically revising for medical content. GB was involved in study design, conception and coordination. SLF was involved in study design, conception and coordination. MA was involved in study design, conception and coordination. All authors have read and approved the manuscript for publication.
